# Research on the control strategy of DC microgrids with distributed energy storage

**DOI:** 10.1038/s41598-023-48038-z

**Published:** 2023-11-23

**Authors:** Qiang Li, Feng Zhao, Li Zhuang, Qiulin Wang, Chenzhou Wu

**Affiliations:** 1 State Grid Information and Telecommunication Co., Ltd., Beijing, 102211 China; 2 FuJian YiRong Information Technology Co. Ltd, Fuzhou, 350001 Fujian China

**Keywords:** Electrical and electronic engineering, Energy grids and networks

## Abstract

As a supplement to large power grids, DC microgrids with new energy access are increasingly widely used. However, with the increasing proportion of new energy in DC microgrids, its output fluctuations directly affect the overall stability of the microgrids. Distributed energy storage can smooth the output fluctuation of distributed new energy. In this paper, an AC-DC hybrid micro-grid operation topology with distributed new energy and distributed energy storage system access is designed, and on this basis, a coordinated control strategy of a micro-grid system based on distributed energy storage is proposed. To maintain the voltage stability of the DC bus and make each station have the power-sharing ability, the AC/DC flexibly interconnected converter should adopt two control strategies. The power can flow bidirectional in the power scheduling and distribution of the energy storage station; At the same time, different power distribution schemes will generate different scheduling costs. To optimize the operation of energy storage power stations, an improved particle swarm optimization algorithm is adopted in this paper to optimize the scheduling task allocation scheme. The optimization objective is the lowest scheduling cost, to realize the optimal scheduling of energy storage power stations. In this paper, based on a Matlab/Simulink environment, a microgrid system based on an AC-DC hybrid bus is built. The simulation results verify the effectiveness of the proposed microgrid coordinated control strategy.

## Introduction

With the development and progress of society, the power load increases rapidly, especially the DC load represented by power electronic equipment^1–5^, and the user's demand for power quality and power supply reliability is more diversified, AC system in the face of a series of new challenges show more and more deficiencies. DC technology provides a new way to solve the above problems. DC system comes back to the stage with increasingly mature power electronics technology and shows unique advantages such as high power quality^6–8^, large power supply capacity, small line loss, convenient distributed energy access, no reactive power compensation equipment, etc., which has attracted more and more attention from researchers in recent years. With the rapid development of DC microgrids, more and more researchers realize the important role of user-side distributed energy storage in DC microgrids. On the one hand, due to the volatility and intermittency of wind and solar energy, the output power of the distributed power supply is greatly affected by environmental factors. The stability and reliability of distributed power supply are poor when it is directly used for user-side power supply. Distributed energy storage can greatly improve the power quality and reliability of distributed power supply^9,10^. On the other hand, there is a certain contradiction between distributed power generation and user power consumption in the time dimension. User-side energy storage can reconcile the contradiction between the two sides and improve the power generation efficiency of distributed power supply.

Due to the current development limitations, the user-side distributed energy storage configuration mode in the DC microgrid is extensive, and the types of energy storage are relatively simple. The potential application value of energy storage needs to be explored urgently. The traditional distributed user-side distributed energy storage control can only provide energy storage and supplement the local distributed power supply. It is unable to interact with distributed power supply, DC low-voltage distribution systems, and different types of low-voltage DC loads. Therefore, aiming at the system architecture and configuration optimization of user-side distributed energy storage, the proposed user-side distributed energy storage group control strategy can provide a comprehensive technical reserve for user-side distributed energy storage system design, operation maintenance, fault handling, and other aspects, and provide technical support for DC microgrid.

In the paper^11^, the direct current voltage is kept stable when the input voltage depth falls; In literature^12^, power routers are used to optimize the distribution network and effectively isolate the disturbance. Aiming at the problem of AC grid-connection of distributed power supply; In literature^13^, the grid-connection performance of distributed power supply is improved by adding reactive power compensation device, but the problem of restricting active power transmission after large-scale new energy access needs to be solved. Literature^14^ proposes an active damping control technology for DC microgrids based on state feedback. The converter's duty ratio is designed by taking the oscillating voltage and current of the system as the feedback, and the pole assignment method is adopted to optimize the relevant control parameters. Literature^15^ proposes an active damping method based on the feedforward of the DC of a grid-connected interface converter. The outlet electricity of the converter is fed forward through the high-pass filtering link to the outer voltage loop to compensate for system damping without changing the stable operating point of the system. Literature^16^ analyzed the influence of constant power load on the stability of DC microgrids. By introducing a low-pass filtering link into the drooping link, the output impedance of the system power supply was effectively reduced and the system stability margin was improved. At the same time, the protection of the power system is also very important^17^, Article^18^ analyzes the important role of electric vehicles in microgrids.

In this paper, an AC-DC hybrid micro-grid operation topology with distributed new energy and distributed energy storage system access is designed, and on this basis, a coordinated control strategy of a micro-grid system based on distributed energy storage is proposed to maintain the voltage stability of the DC bus, so that each station has the ability of mutual power exchange, and power can flow bidirectional in the power scheduling and distribution of the energy storage station.

To optimize the operation of energy storage power stations, this paper adopts the improved particle swarm optimization algorithm to optimize the scheduling task allocation scheme. The optimization objective is the lowest scheduling cost, to realize the optimal scheduling of energy storage power stations. In this paper, based on the Matlab/Simulink environment, a microgrid system based on an AC-DC hybrid bus is built. The simulation results verify the effectiveness of the proposed microgrid coordinated control strategy.

## Establishment of a distributed energy storage model

### DC-DC converter suitable for DC microgrid

**Figure 1:**
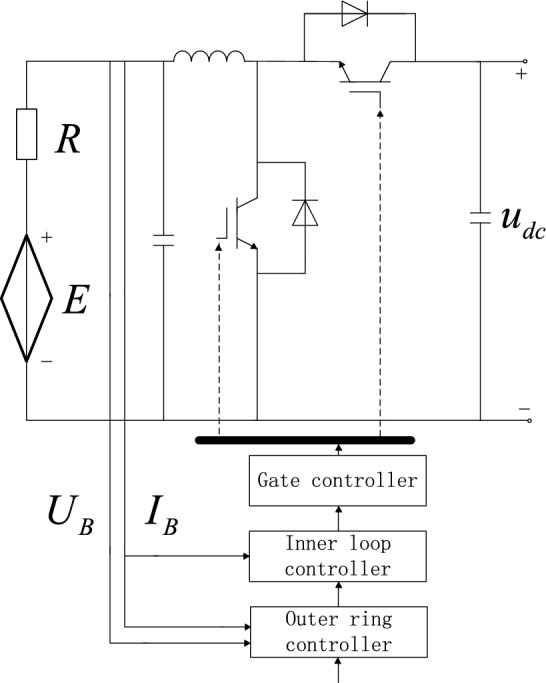
Grid connection topology of distributed energy storage.

Distributed energy storage needs to be connected to a DC microgrid through a DC-DC converter^13,14,16,19^, to solve the problem of system stability caused by the change of battery terminal voltage and realize the flexible control of distributed energy storage (Fig. 1).

In the figure, the bidirectional DC-DC converter adopts the current reversible chopper circuit, and the charge and discharge are realized through the Buck and Boost operating modes of the DC-DC converter.

**Figure 2:**
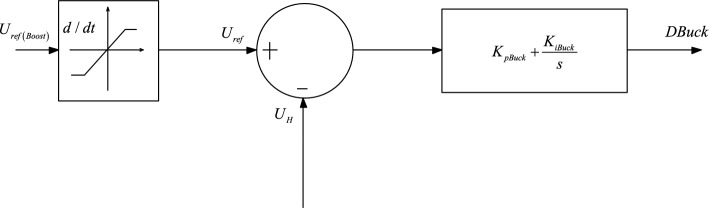
Typical charging control of a DC-DC converter.

When there is a power deficit in the DC microgrid, $P_{ref}$ the distributed energy storage system releases power. Figure 2 shows the typical control structure of the system controller based on the internal power of the microgrid.

**Figure 3:**
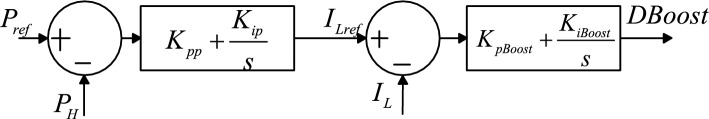
Typical discharge control of a DC-DC converter.

The difference between the required energy generation of distributed energy storage with a fixed gap and the actual output power is adjusted by PI to output the reference value of the distributed energy storage discharge current^15,20–23^. The power control signal is output by the difference between the value and the actual current again controlled by PI. Figure 3 is the Typical discharge control of a DC-DC converter.

**Figure 4:**
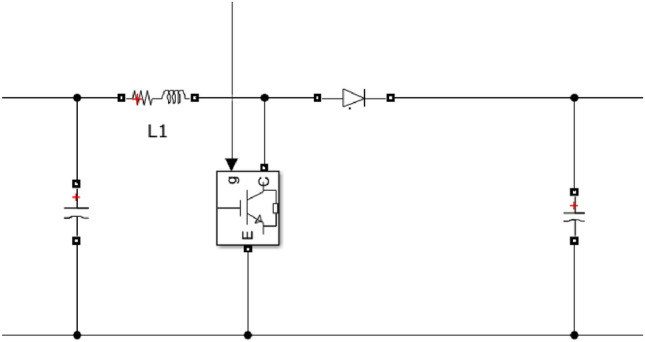
Simulation diagram of DC-DC converter.

**Figure 5:**
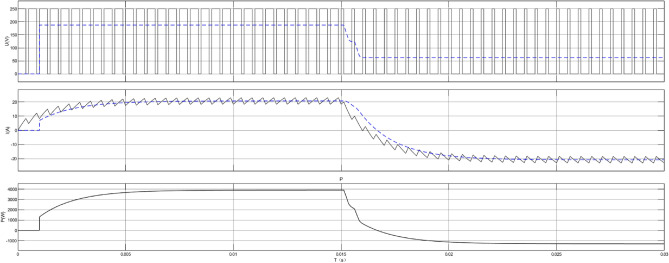
Waveform modulation diagram.

In this paper, relevant parameters of DC-DC converters applicable to DC microgrids in the topology are defined, as shown in Table 1, and a simulation model is established based on MATLAB for DC-DC converters, with a stable output of voltage and power. Figure 4 shows the converter principle diagram, and Fig. 5 shows the converter waveform modulation diagram.

**Table 1:** Typical parameters of the DC-DC converter.

Duty cycle	0.5
MPPT Time window (s)	200
PWM frequency (Hz)	5 000
Input voltage (V)	273
Output voltage (V)	750

### Analysis of the charged state of distributed energy storage

The operating characteristics of distributed energy storage are affected by the battery's state, temperature, and other factors, so it is necessary to study the relevant parameters before giving the equivalent circuit of distributed energy storage^24–28^. The state of charge is a parameter used to characterize the residual capacity of distributed energy storage^29–31^, which is defined as the ratio of the residual capacity of distributed energy storage to the standard capacity:

1 $$SOC = \frac{{Q_{r} }}{C}$$

where, $Q_{r}$: the remaining capacity of the battery; C: The standard capacity of the capacitor.

Assuming that the charged state of distributed energy storage at the initial time of distributed energy storage is, the estimated value can be calculated by the formula

2 $$SOC = SOC_{0} - \frac{{Q_{e} }}{C}$$

where, $Q_{e}$: Discharge quantity of distributed energy storage during operation. If the charging and discharging efficiency of the battery is $\eta$,

3 $$Q_{e} = \int_{0}^{t} \eta l_{B} \left( \tau \right)d\tau$$

Then the potential of the equivalent controlled voltage source can be calculated by the following formula

4 $$E_{m} = E_{0} - K\frac{{C_{\max } }}{{C_{\max } - Q_{e} }} + A\exp \left( { - BQ_{e} } \right)$$

where, $E_{0} (V)$ is the no-load potential of distributed energy storage, which $C_{\max }$ can be calculated through the actual state of the battery; Is the maximum capacity of distributed energy storage; The coefficient $A\left( V \right)$,$K\left( V \right)$, and are constants, which can be obtained by fitting the discharge characteristic curve of distributed energy storage.

## Research on optimal operation strategy of charge and discharge of energy storage system considering battery life

In the power dispatching and distribution of energy storage stations, different power distribution schemes will produce different dispatching costs. To optimize the operation of the energy storage power station, it is necessary to optimize the scheduling task allocation scheme. In this paper, the Particle Swarm Optimization (PSO) algorithm is adopted to optimize the scheduling task allocation scheme^32–34^. The optimization goal is the lowest scheduling cost, to realize the optimal scheduling of energy storage power stations.

When a particle swarm optimization algorithm is used to solve optimization problems, Each particle has its position and velocity, and a fitness value determined by the fitness function. The process of each iteration is not completely random, and if a better solution is found, it will be used as a basis to find the next solution. Initialize PSO into a group of (random solutions). In each iteration, two extreme points will be generated: the first is the best solution found by the particle itself, and the other is the best solution found by the whole population, which is called the global extreme point. After the above two extreme points are found, the particle swarm is iteratively updated according to Eqs. (5) and (6).

5 $$v_{id}^{k + 1} = v_{id}^{k} + c_{1} {\text{rand}}_{1}^{k} \left( {{\text{pbest}}_{id}^{k} - x_{id}^{k} } \right) + c_{2} {\text{rand}}_{2}^{k} \left( {{\text{gbest}}_{id}^{k} - x_{id}^{k} } \right)$$

6 $$x_{id}^{k + 1} = x_{id}^{k} + v_{id}^{k + 1}$$

Where, $v_{id}^{k}$ represents the velocity of particle I in dimension d in the KTH iteration; And represents the acceleration coefficient (or learning factor), which regulates the maximum stride length to the global best particle and the individual best particle respectively. If it is too small, the particle may be far away from the target region; if it is too large, it will suddenly fly to or fly over the target region. ${\text{rand}}_{1}^{k}$ And ${\text{rand}}_{2}^{k}$ represents a random number between 0 and 1; $x_{id}^{k}$ Represents the current position of particle I in dimension d in the KTH iteration; ${\text{pbest}}_{id}^{k}$ Represents the position of individual extreme point of particle I in dimension d; ${\text{gbest}}_{id}^{k}$ Represents the position of the global extreme point of the whole group in dimension.

To apply the particle swarm optimization algorithm to the optimal scheduling of an energy storage power station, the parameters in the particle swarm optimization algorithm need to correspond to the relevant scheduling parameters in an energy storage power station. "Particle" corresponds to the scheduling task allocation scheme in the energy storage power station; "Fitness function" corresponds to the scheduling cost calculation function in the energy storage power station.

The optimization objective of this project is the lowest dispatching cost of an energy storage power station within a unit dispatching period. The task allocation scheme with the lowest dispatching cost within a unit period is sought through the particle swarm optimization algorithm, and the optimization objective is shown in Eq. (7).

7 $$\min C = C_{fix} + C_{loss} + C_{f}$$

In Eq. (7), C represents scheduling cost; Cfix stands for operation and maintenance cost; Closs is the cost of wear and tear. Cf stands for a fixed cost.

Energy storage power stations will be restricted by some factors during operation, which should be taken into account when applying particle swarm optimization algorithms for optimization, specifically including the following three points.

The dispatching tasks of each battery system should be within the rated power range. That is, when the power station system discharges, the assigned discharge power should be within its rated discharge power; When charging a power station system, the discharge power assigned to it should be within its rated charging power, as shown in Eq. (8).

8 $$- \hat{P}_{dis,i} \le P_{i}^{{}} \le \hat{P}_{chg,i}$$

Formula (8), $\hat{P}_{dis,i}$ denotes the rated discharge power of the I-th battery system, unit kW; $\hat{P}_{chg,i}$ Indicates the rated charging power of the I-th battery system, in kW.

The SOC of the battery system is within the set range. The outgoing power value when the battery system reaches its own SOC lower limit and the received power value, when it reaches its own SOC upper limit, is calculated, and the dispatching task assigned by the battery system is limited to these two dispatching power values, as shown in Eq. (9).

9 $${\text{SOC}}_{i}^{\min } \le {\text{SOC}}_{i}^{{}} \le {\text{SOC}}_{i}^{\max }$$

In Formula (9), SOCi represents the SOC of the I-th battery system. SOCimin indicates the lower SOC limit of the I-th battery system. SOCimax represents the upper SOC of the I-th battery system.

When scheduling tasks are within the acceptable range of the energy storage power station, the sum of scheduling tasks of all battery systems should be equal to the total scheduling tasks, as shown in Eq. (10).

10 $$P_{total} = \sum\limits_{i = 1}^{n} {P_{i} }$$

In Formula (10), Ptotal represents the total scheduling task (unit: kW). Pi Indicates the dispatching power of battery system I (unit: kW).

The input of the optimization algorithm in this paper includes scheduling tasks and fixed and variable parameters of the energy storage station. Scheduling tasks include total scheduling tasks and scheduling time, and fixed parameters include the number of battery systems in the energy storage station, rated voltage, rated power, rated capacity, rated electricity, fixed construction cost, and charging and discharging efficiency. Variable parameters include the current SOC, current SOH, cumulative time, and cumulative cycles of each battery system in the energy storage station. The output of the optimization algorithm includes SOC, SOH, cumulative time, the cumulative number of cycles, optimal allocation scheme, and corresponding scheduling cost of each battery system after the simulation schedule.

**Figure 6:**
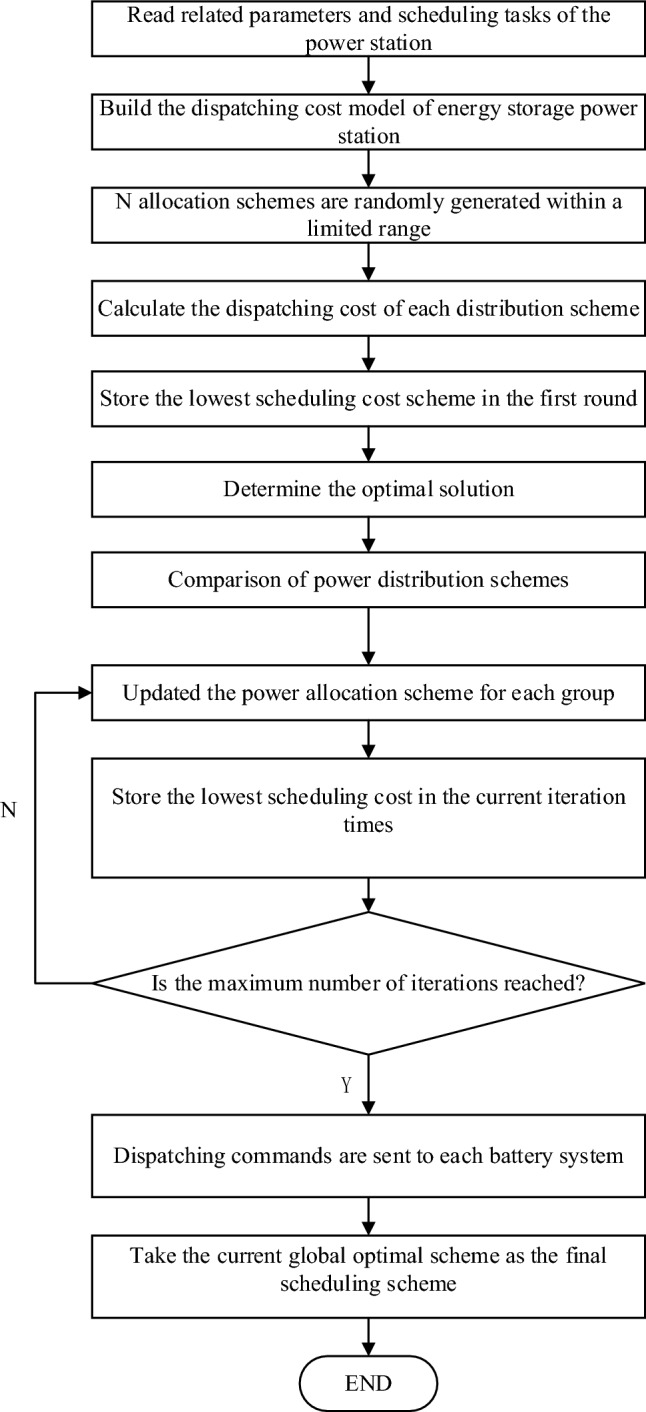
Flow chart of optimized scheduling policy.

The flow chart of the optimal scheduling strategy for energy storage power stations is shown in Fig. 6.

The specific steps are as follows:

1. Read related parameters. The parameters include the rated power, rated capacity, current SOC, and current SOH of each battery system.
2. N populations are randomly generated. That is, N power distribution schemes are used to allocate power within the capacity range of each battery system, thus limiting the power distribution range.
3. Selection of individual optimal solution. By comparing the dispatching cost of the current N power distribution schemes, the scheme with the lowest dispatching cost is selected as the optimal scheme under the current iteration times, namely, the individual optimal solution.
4. Solution update. Solutions under the same number of iterations “converge” to the current individual optimal solution, and the “converge” speed is specified by the algorithm. The current N allocation schemes are iteratively updated to generate N new allocation schemes.
5. Selection of global optimal solution. In the updated allocation scheme, the scheme with the lowest scheduling cost is selected and updated into the current global optimal solution. When the number of iterations reaches the set value, the current global optimal solution is the final global optimal solution. Otherwise, go back to Step 4 to continue the iteration.
6. Store-related parameters. Update the parameters changed after each dispatch, including the SOC, SOH, cumulative duration, and cumulative cycles of the battery system.

In this project, the particle swarm optimization algorithm is applied to the optimal scheduling of energy storage power stations. The lowest unit cycle scheduling cost is taken as the optimization objective, and relevant limiting factors are taken into account. The optimal allocation scheme is obtained by the algorithm to realize the optimal scheduling of the energy storage power station.

To verify the feasibility of the proposed power distribution and scheduling scheme for the energy storage station considering the SOH attenuation of the battery system, simulation experiments are carried out based on the established energy storage station scheduling cost model, and comparison and analysis are carried out with the equal proportion distribution scheme.

- (1) Simulation parameter setting

The schematic diagram of the energy storage station in this case is shown in Fig. 1, where the number of battery systems n is 4, that is, the energy storage station in this case contains four independent battery systems. The relevant parameters of the energy storage power station are shown in Table 2. Table 2 shows the Relevant parameters of the energy storage power station in the case.

**Table 2:** Relevant parameters of energy storage power station in the case.

Correlation parameter	B1	B2	B3	B4
Rated voltage (kV)	0.4	0.4	0.3	0.2
Rated power (kW)	100	150	100	150
Rated electric quantity (kWh)	150	100	100	100
Charging efficiency	95%	95%	95%	95%
Discharge efficiency	95%	95%	95%	95%
Initial SOC	55%	60%	55%	60%
Initial SOH	100%	100%	100%	100%

In the simulation scheduling process, in addition to the above parameters, the following constraints should also be considered: (1) Limitations of battery system SOC. To protect the battery system from working within a reasonable range, the allowable SOC range of each independent battery system was set to 20–100% in the simulation schedule. (2) Limitations of battery system SOH. Considering that the battery's SOH attenuates to a certain value, its performance parameters will change to a certain extent, which will affect its efficiency and even become a safety hazard. Therefore, the lower limit of SOH in this case simulation is set at 50%, that is, when the SOH of a battery system attenuates to 50%, it is considered that the battery system cannot continue to work.

### Simulation design

The input parameters of the proposed algorithm include the rated power, rated capacity, and other basic parameters of the energy storage station, as well as scheduling tasks and scheduling time. The output parameters of the algorithm include the optimal power scheduling scheme, the loss cost generated by the optimal scheme, and the change of related parameters after the scheduling of each battery system.

**Figure 7:**
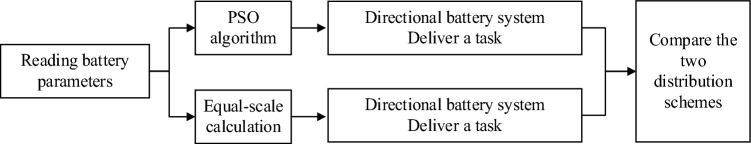
Steps of simulation experiment.

To reflect the superiority of the proposed algorithm, the power distribution method is compared according to the ratio of the actual acceptable maximum power of each battery system, that is, the proportional distribution scheme of scheduling tasks and the PSO algorithm in this paper are compared and analyzed. The schematic diagram of the simulation experiment steps is shown in Fig. 7.

In the simulation experiment, the initial SOH of each battery system is 100%, until the SOH of any battery system attenuates to 50%, and the simulation experiment stops. To make the simulation parameters closer to the actual situation, the dispatching task in the simulation experiment takes the typical working condition of peak and valley cutting, and its dispatching power is successive -100 kW, -200 kW, -400 kW, -300 kW, -200 kW, 200 kW, 300 kW, 400 kW, 200 kW, 100 kW, forming a cycle. The unit scheduling period is set to 5 min, and negative power indicates discharge.

**Figure 8:**
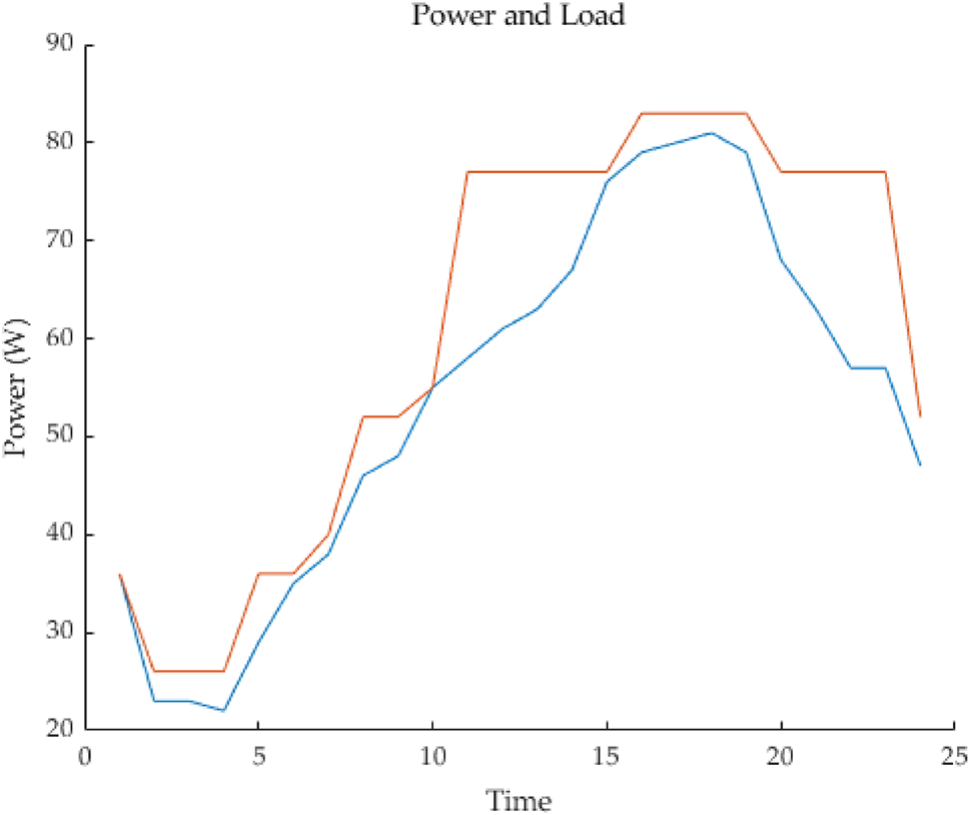
Battery system SOC change curve 8 Battery system SOC change curve.

**Figure 9:**
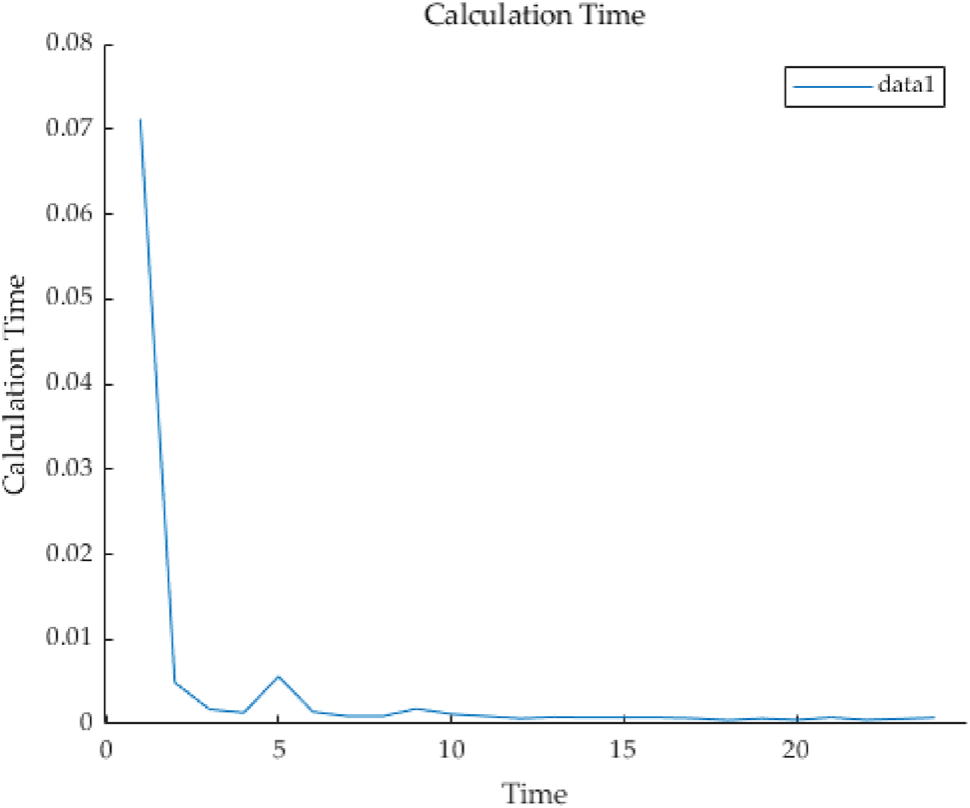
Calculation time of battery system algorithm.

The simulation results of the PSO algorithm and equal proportional distribution are shown in Figs. 8 and 9. First, Fig. 4 shows the SOC change curve of battery system B3. With the increase of the dispatching times of the energy storage power station, the minimum capacity of B3 of the battery system gradually decays, resulting in an increasing range of SOC variation in the case of the same dispatching task, which even reaches the lower limit set by SOC (20%), indicating that the battery system has a large discharge depth in the simulation…

## Control strategy analysis of four regional flexible interconnection systems

**Figure 10:**
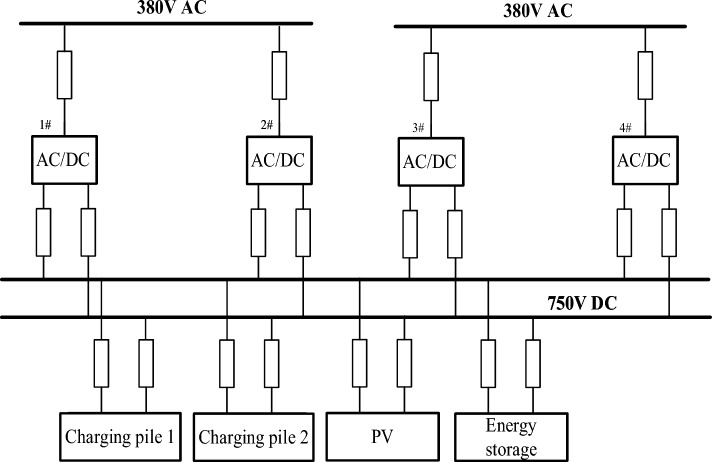
Flexible interconnection topology of four zones.

Each element in Fig. 10 shows that 1#, 2#, 3#, and 4# are 630kVA*2 + 1000kVA*2 platform transformers; Four AC/DC power flow controllers with a rated power of 250 kW; Two 60 kW DC quick charging piles; Energy storage battery 250kWh, rated power 100 kW; The rated PV installed power is 100 kW. The biggest difference between this topology and other conventional topologies is that the DC microgrid power supply is used in all power supply stations, and the topology contains both photovoltaic systems and power storage systems, which can optimize the energy flow.

**Figure 11:**
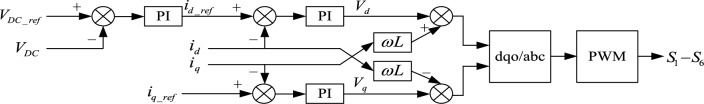
AC/DC constant DC voltage control.

**Figure 12:**
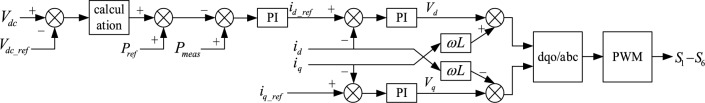
AC/DC power-DC voltage droop control.

**Figure 13:**
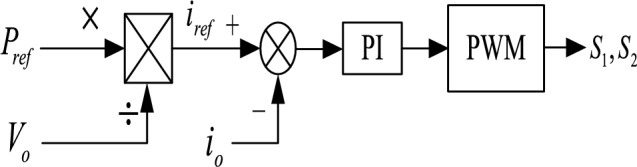
Constant power control of electric vehicle charging pile.

**Figure 14:**
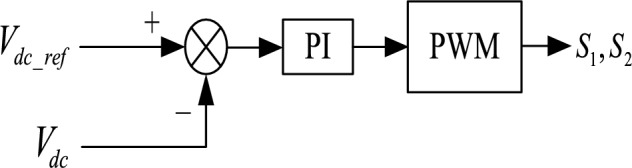
Constant voltage control of energy storage battery.

**Figure 15:**
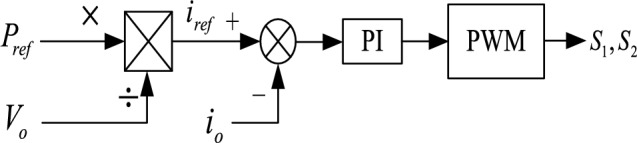
Constant power control of energy storage battery.

**Figure 16:**
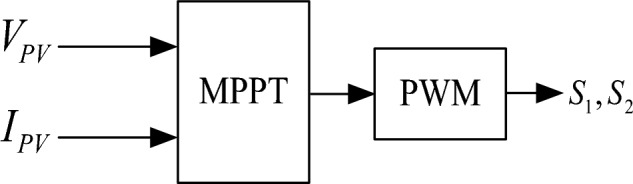
Photovoltaic MPPT control.

To maintain the voltage stability of the DC bus and make each station have the power-sharing ability, the AC/DC flexible interconnected converter should adopt two control strategies, namely constant DC voltage control and power-DC voltage droop control. The power can flow bidirectional. The block diagrams of the two control strategies are shown in Figs. 11 and 12 respectively. Electric vehicle charging piles adopt constant power control and can V2G power bidirectional flow. The Constant power control of the electric vehicle charging pile is shown in Fig. 13. The energy storage battery adopts two control strategies, constant DC voltage control, and constant power control, and the power can flow bidirectional. The block diagram of the control strategy is shown in Figs. 14 and 15. MPPT maximum power tracking control is adopted for photovoltaic power generation, as shown in Fig. 16.

Table 3 is the Simulation test conditionsThe simulation test conditions are set as follows, Table 4 shows the simulation values.

**Table 3:** Simulation test conditions.

Device name	Control policy	Power direction
1#AC/DC	Constant DC voltage	DC power grid to AC power grid
2#AC/DC	droop	DC power grid to AC power grid
3#AC/DC	droop	Ac power grid to DC power grid
4#AC/DC	Constant DC voltage	Ac power grid to DC power grid
1# Charging pile	Constant power	DC grid to electric vehicles
2# Charging pile	Constant power	DC grid to electric vehicles
PV	MPPT	Photovoltaic to DC grid

**Table 4:** Simulation concrete value.

Device name	Simulation values
1# DC	750 V
2# DC	750 V
3# DC	750 V
4# DC	750 V
1# AC	380 V
2# AC	380 V
3# AC	380 V
4# AC	380 V

**Figure 17:**
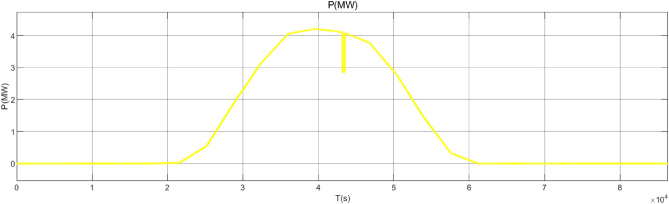
Photovoltaic output active work.

**Figure 18:**
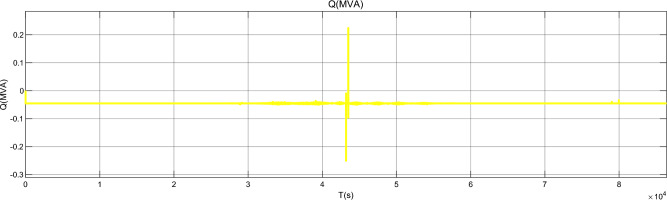
Photovoltaic output reactive power.

**Figure 19:**
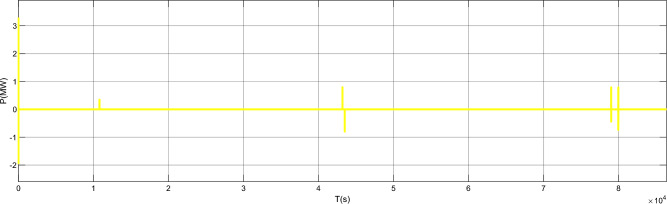
Regulation of active power of charging piles.

**Figure 20:**
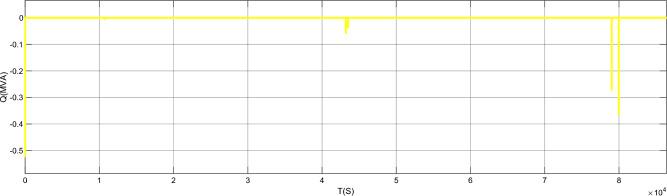
Reactive power Regulation of charging pile.

**Figure 21:**
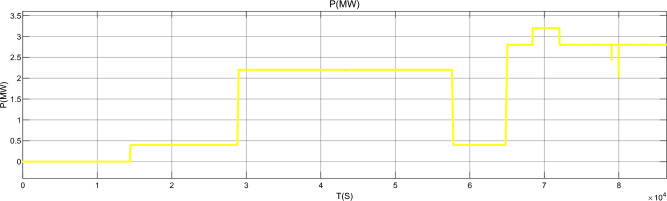
Active Charging of charging pile.

**Figure 22:**
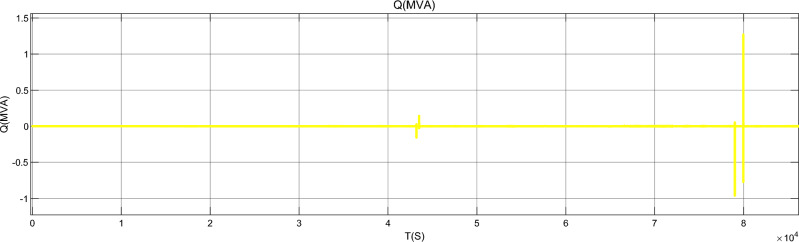
Reactive Charging of charging pile.

**Figure 23:**
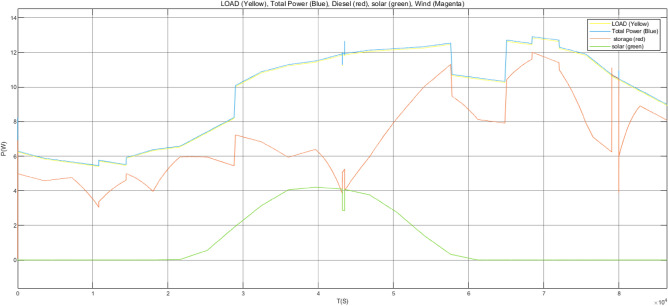
Sequence diagrams of PV, energy storage, and load active power in different time sequences.

Figure 17 is the active power of photovoltaic output, Fig. 18 is the reactive power of photovoltaic output, Fig. 19 is the active power Regulation of the Charging pile, Fig. 20 is the reactive power Regulation of the Charging pile, Fig. 21 is the active power charging of the charging pile, Fig. 22 is the reactive power charging of the charging pile, Fig. 23 is the photovoltaic and energy storage of different time sequences, Load active power sequence diagram.

**Figure 24:**
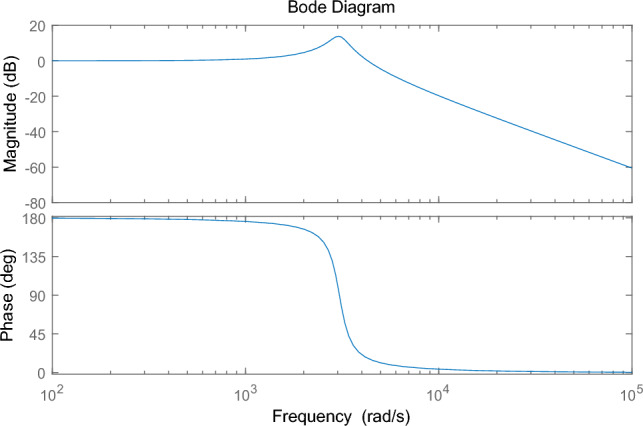
bode diagram of a transfer function of output voltage disturbance and input voltage disturbance.

**Figure 25:**
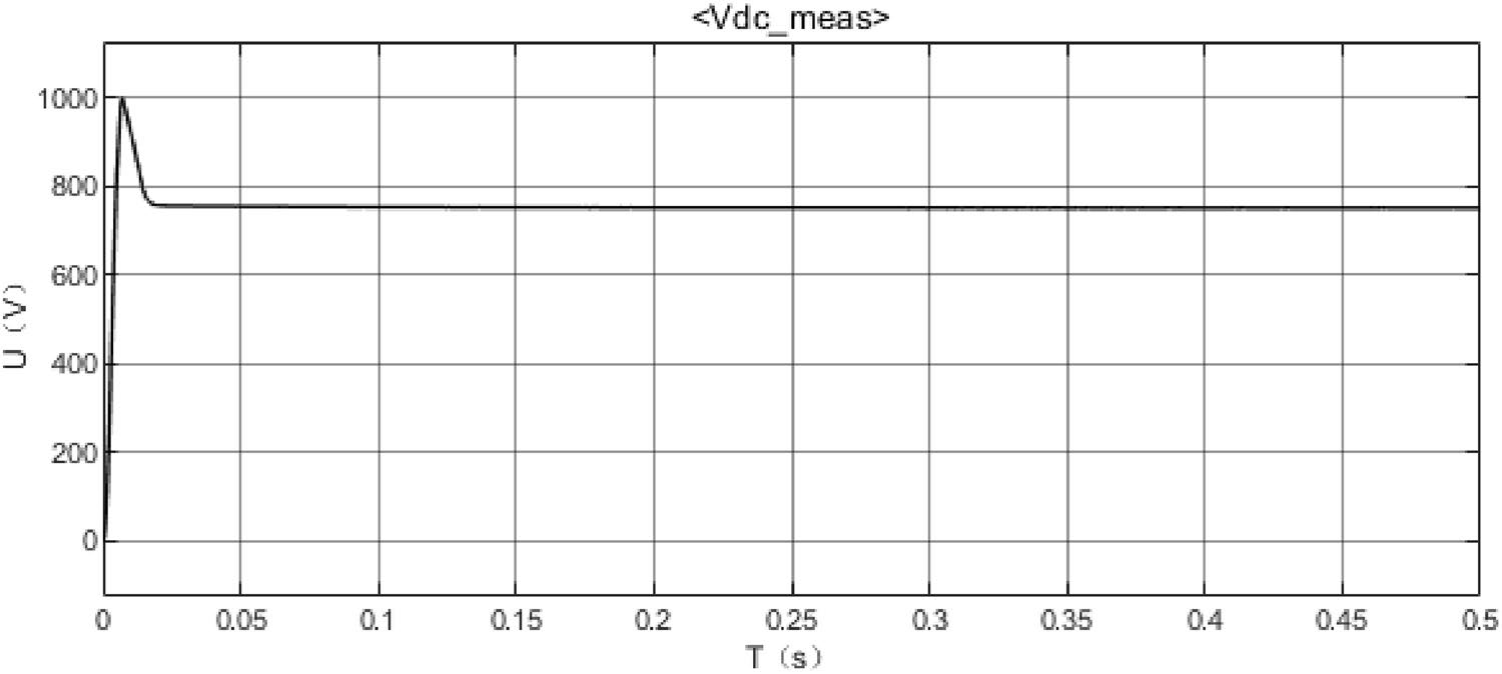
DC voltage of the DC bus node.

**Figure 26:**
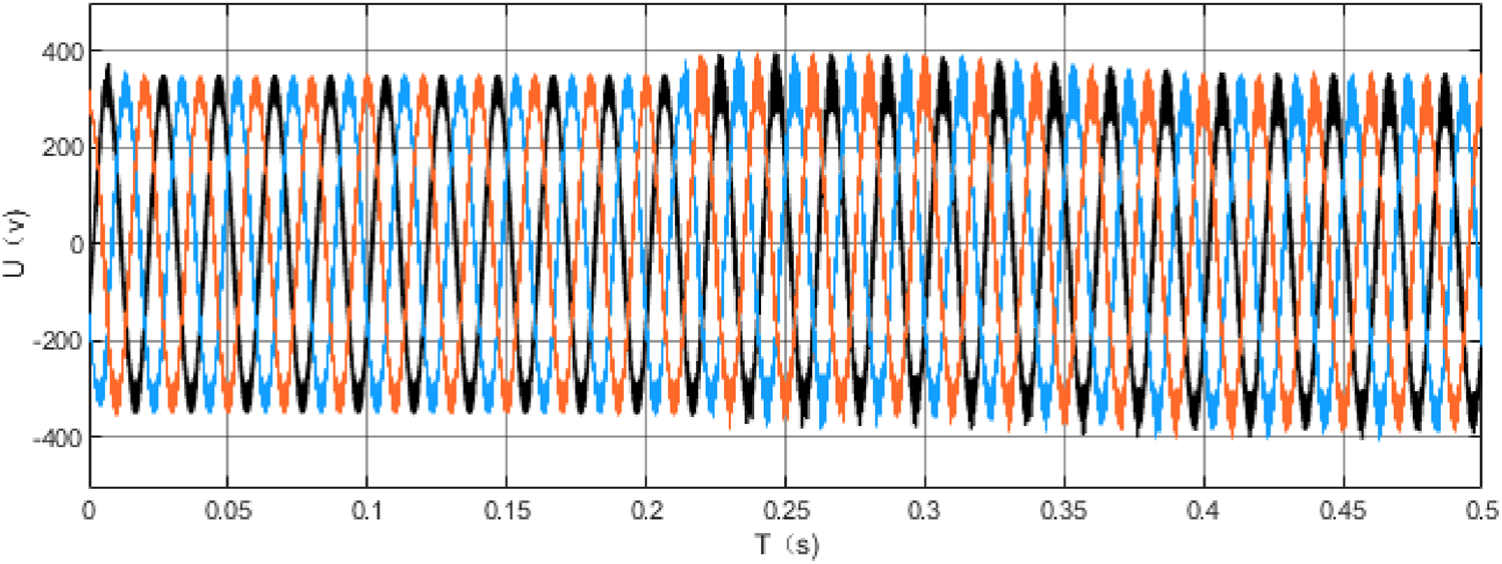
AC bus node AC voltage.

MATLAB is used to draw the bode diagram of the transfer function of the output voltage disturbance and the input voltage disturbance, as shown in Fig. 24, Fig. 24 is bode diagram of a transfer function of output voltage disturbance and input voltage disturbance, It embodies the amplitude-frequency characteristics and phase-frequency characteristics of the whole system frequency, Fig. 25 shows the voltage of the DC bus node, Fig. 26 shows the AC bus node AC voltage.

The simulation results show that the energy storage device can effectively stabilize the voltage of the DC bus when operating in constant DC voltage mode. Now, when an AC/DC flexible interconnected converter adopts constant DC voltage control, the voltage comparison between the DC bus without the energy storage device and the one with the energy storage device is shown. The DC bus voltage steady-state fluctuation error of the DC bus voltage equipped with the energy storage device is smaller, which proves that the energy storage device can effectively stabilize the DC bus voltage. The working mode of the energy storage device is constant power mode, the power of the energy storage device is set, and the direction is from the energy storage device to the DC power grid. MATLAB is used to draw the bode diagram of the transfer function of the output voltage disturbance and the input voltage disturbance, which verifies the stability of the system.

## Conclusions

In this paper, an AC-DC hybrid micro-grid operation topology with distributed new energy and distributed energy storage system access is designed, and on this basis, a coordinated control strategy of the micro-grid system based on distributed energy storage is proposed to maintain the voltage stability of the DC bus, so that each station has the ability of mutual power exchange, and power can flow bidirectional in the power scheduling and distribution of the energy storage station. To optimize the operation of the energy storage power station, this paper adopts the improved particle swarm optimization algorithm to optimize the scheduling task allocation scheme. The optimization objective is the lowest scheduling cost, to realize the optimal scheduling of energy storage power stations. In this paper, based on the Matlab/Simulink environment, a microgrid system based on an AC-DC hybrid bus is built. The simulation results verify the effectiveness of the proposed microgrid coordinated control strategy.

## Data Availability

The data that support the findings of this study are available from[State Grid Information & Telecommunication Co., Ltd., Beijing, li_qiang_xc@163.com] but restrictions apply to the availability of these data, which were used under license for the current study, and so are not publicly available. Data are however available from the authors upon reasonable request and with permission of [State Grid Information & Telecommunication Co., Ltd., Beijing li_qiang_xc@163.com]. Anyone who would like data from this study should contact liqiang, li_qiang_xc@163.com.
